# Case report: Duodenal obstruction caused by gastroduodenal artery pseudoaneurysm with hematoma: an unusual case and literature review

**DOI:** 10.3389/fmed.2023.1198378

**Published:** 2023-06-22

**Authors:** Yan-Yuan Zhou, Shao-Chung Wang, Chen-June Seak, Shu-Wei Huang, Hao-Tsai Cheng

**Affiliations:** ^1^School of Medicine, College of Medicine, Chang Gung University, Taoyuan, Taiwan; ^2^Department of Medical Imaging and Intervention, New Taipei Municipal Tucheng Hospital, Chang Gung Medical foundation, New Taipei City, Taiwan; ^3^Department of Emergency Medicine, Linkou Medical Center, Chang Gung Memorial Hospital, Taoyuan, Taiwan; ^4^Department of Emergency Medicine, New Taipei Municipal TuCheng Hospital, New Taipei City, Taiwan; ^5^Division of Gastroenterology and Hepatology, Department of Internal Medicine, New Taipei Municipal TuCheng Hospital, New Taipei City, Taiwan; ^6^Graduate Institute of Clinical Medicine, College of Medicine, Chang Gung University, Taoyuan, Taiwan

**Keywords:** duodenal obstruction, gastroduodenal artery, pseudoaneurysm, transarterial embolization, liquid embolic agent, case report

## Abstract

Visceral artery pseudoaneurysm is a rare disease that most commonly occurs in male patients in their 50s, with gastroduodenal artery (GDA) pseudoaneurysm accounting for only 1.5% of these. The treatment options generally include open surgery and endovascular treatment. In 40 cases of GDA pseudoaneurysm from 2001 to 2022, endovascular therapy was the mainstay of treatment in 30 cases, and most of them (77%) were treated by coil embolization. Our case report describes a 76-year-old female patient with a GDA pseudoaneurysm, which was treated by endovascular embolization using liquid embolic agent N-butyl-2-cyanoacrylate (NBCA) alone. This is the first time this treatment strategy has been used for GDA pseudoaneurysm. We demonstrate a successful outcome with this unique treatment. The successful experience of our case may provide a new treatment strategy for this rare disease.

## 1. Introduction

Visceral artery aneurysm, including true aneurysm and pseudoaneurysm, is a rare disease, with an incidence of 0.01–0.2% (1). A true aneurysm is the dilation of all three layers of the vessel wall and is often caused by atherosclerosis as well as hypertension (2). A pseudoaneurysm is blood accumulation surrounded by fibrous tissue, whose etiologies are inflammation, vasculitis, iatrogenic trauma, or infection resulting in blood extravasation *via* this damage into the space between the tunica media and tunica adventitia (3). There is usually a periarterial hematoma surrounding the pseudoaneurysm (4).

The most frequent sites of visceral artery aneurysm, in the order of the most to least common, are the splenic (60%), hepatic (20%), and superior mesenteric arteries (5.5%), with the gastroduodenal artery accounting for only 1.5% of cases (3). Traditionally, the treatment of visceral artery aneurysm is *via* open surgery. However, in the last two decades, endovascular treatment has become the mainstay therapy in hemodynamically stable patients, with coil embolization being the most commonly used endovascular therapy. In this case report, we present a special case of duodenal obstruction caused by GDA pseudoaneurysm and an associated intramural hematoma. We used the liquid embolic agent NBCA, a unique treatment strategy, as our means of endovascular therapy and achieved a successful outcome. A review of relevant medical literature about GDA pseudoaneurysm in the last two decades is also presented in this case report.

## 2. Case report

**Figure 1:**
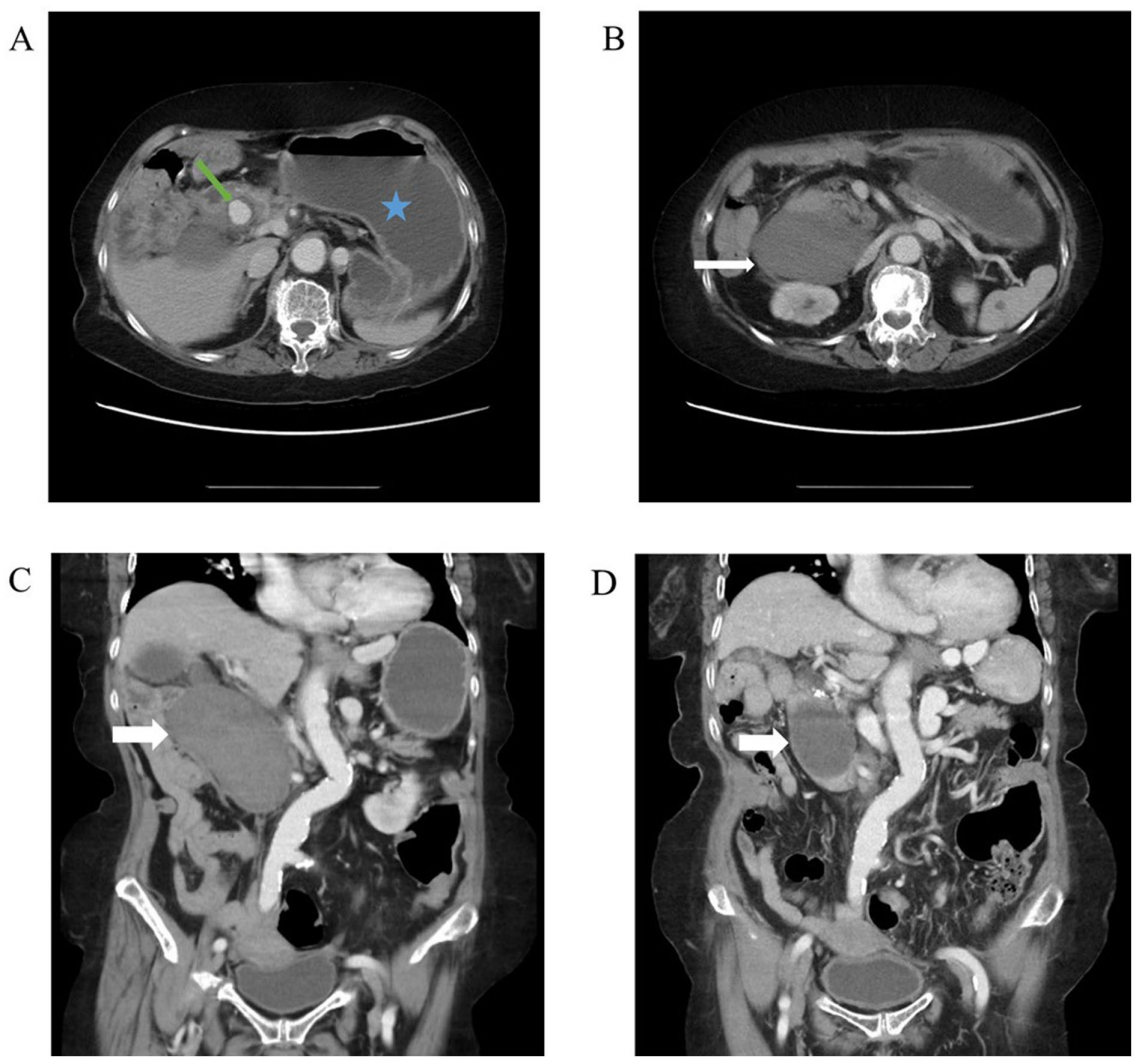
Abdominal CT. **(A)** Axial view of abdominal CT revealed a distended abdomen (blue star) and an enhanced circular lesion around duodenal second portion (green arrow). Due to its location corresponding to gastroduodenal artery, it was more likely to be a pseudoaneurysm than a true aneurysm. **(B)** Axial view of abdominal CT revealed large, non-enhanced mass (white arrow). Given the patient’s hemodynamic stability and absence of signs for gastrointestinal bleeding, it was likely to be an intramural hematoma. **(C)** Coronal view of abdominal CT showed large intramural hematoma (white arrow). **(D)** Coronal view of abdominal CT 3 months after transarterial embolization (TAE) revealed shrinkage of the hematoma (white arrow).

**Figure 2:**
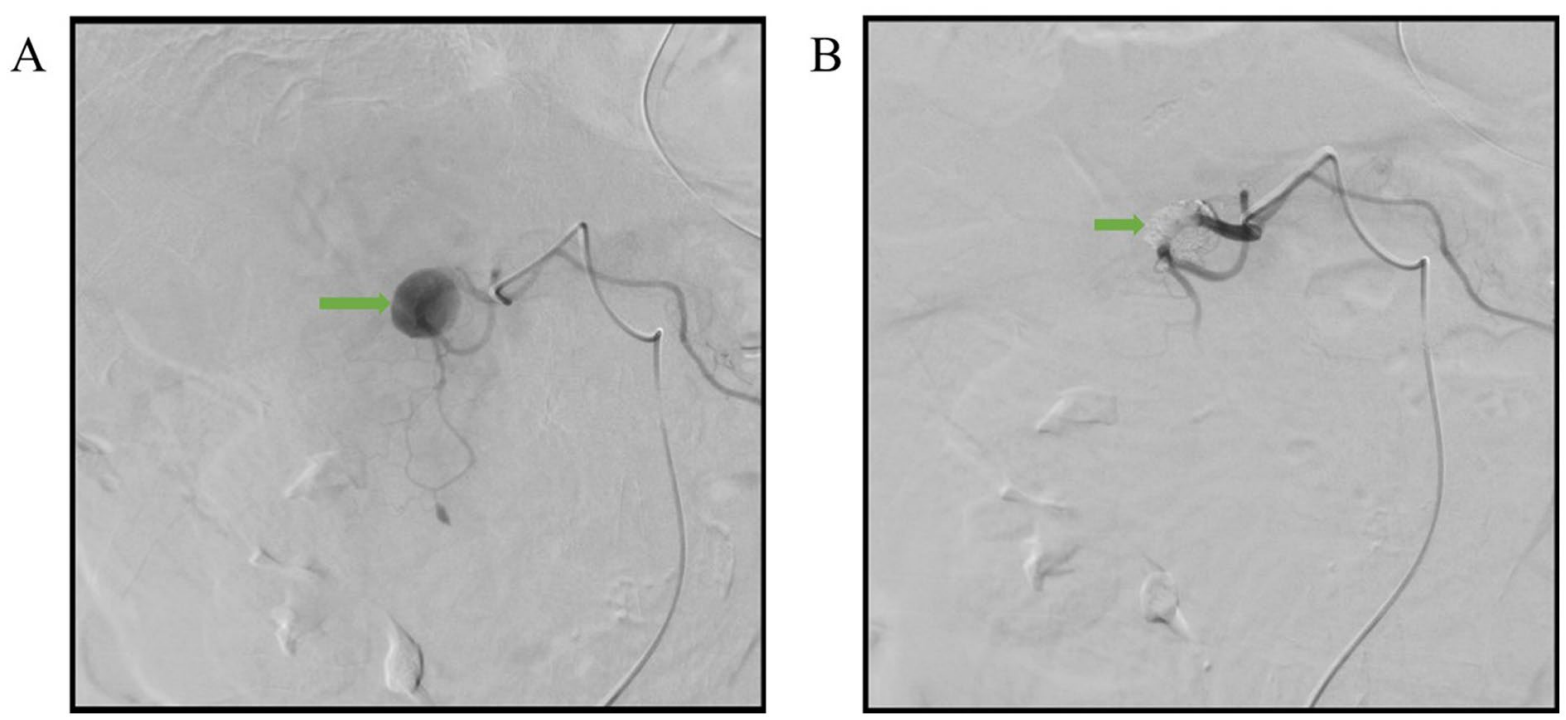
Angiographic images. **(A)** The pseudoanuerysm (arrow) originated from the side branch of proximal gastroduodenal artery. **(B)** Complete embolization of the pseudoaneurysm (arrow) with NBCA.

A 76-year-old female with past histories of a gastric ulcer, chronic superficial gastritis, and morbid obesity came to the emergency department due to fever with abdominal pain for 1 day. The abdominal pain was dull, localized, and persistent in character. Associated symptoms included nausea and vomiting with food content. The patient did not have diarrhea, tarry stool, or bloody stool. The physical examination showed that the abdomen was mildly distended and that there was upper abdominal tenderness. The laboratory findings revealed leukocytosis of 21,700/μL, elevated C-reactive protein level of 123 mg/L, anemia with hemoglobin level of 8.4 g/dL, and electrolyte disturbance with hypokalemia of 3.1 mEq/L. Due to the above findings, intra-abdominal infection was initially suspected. Therefore, contrast-enhanced computed tomography (CT) of the lower chest, abdomen, and pelvis was performed. The CT incidentally demonstrated a pseudoaneurysm with a large intramural hematoma up to 8.8 cm at the second portion of the duodenal wall, the large hematoma compress the duodenal lumen and therefore cause the distention of stomach and esophagus (Figures 1A–C). After discussion with gastroenterologists and general surgeons, transarterial embolization (TAE) for pseudoaneurysm was indicated. The angiography revealed that the pseudoaneurysm originated from the side branch of the proximal gastroduodenal artery (GDA) (Figure 2A), and embolization of both the pseudoaneurysm (Figure 2B) and feeding artery was performed using N-butyl-2-cyanoacrylate (NBCA), one kind of liquid embolic agent.

**Figure 3:**
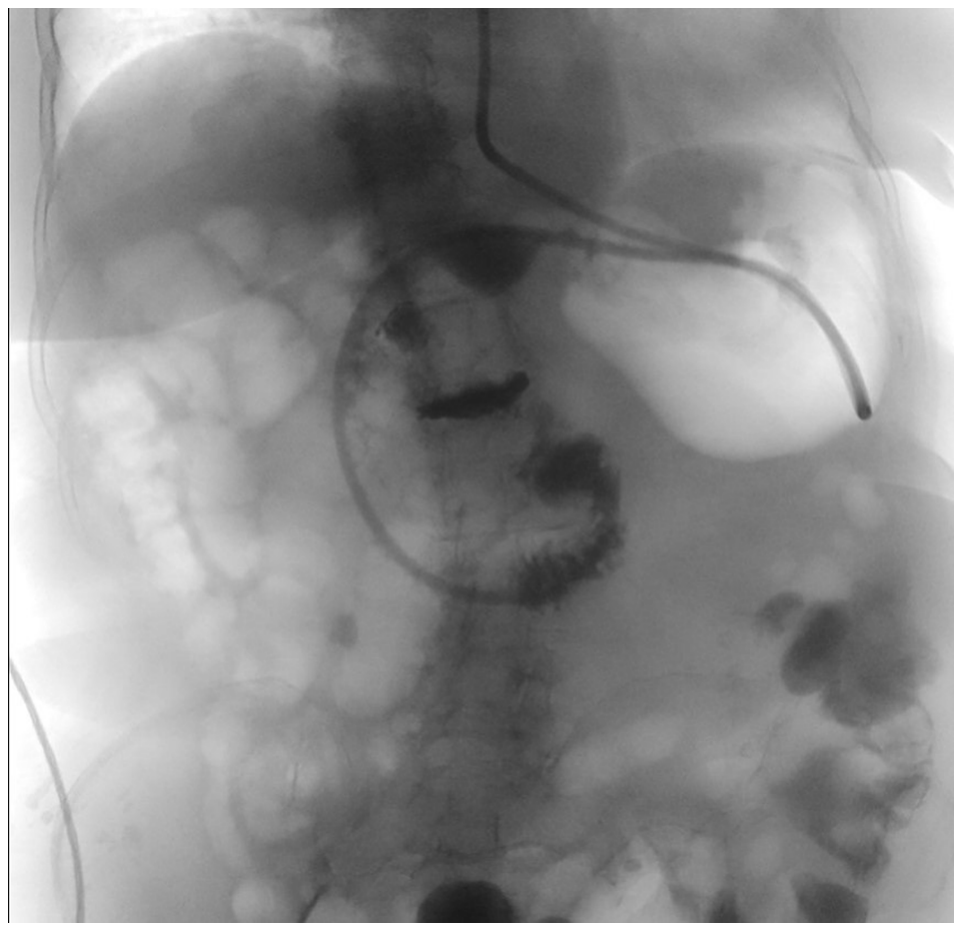
Fluoroscopic image demonstrates placement of nasoduodenal tube.

After TAE, the patient’s condition was stable. The patient was initially fasted except for water and underwent nasogastric tube decompression. Intravenous fluid resuscitation, an antibiotic for infection control, and lansoprazole for long-term gastric ulcer were also given. On day 5 of admission, we placed a nasoduodenal (ND) tube under endoscopic guidance for feeding of nutritional supplements (Figure 3). On day 14 of admission, the patient started to try oral ingestion in addition to ND tube feeding, which was completed smoothly. Hence, she was discharged on day 15. One month after discharge, the patient recovered well when followed-up in the outpatient clinic. Three months after TAE, the follow-up CT image revealed shrinkage of the hematoma to 5.7 cm (Figure 1D).

## 3. Discussion

Visceral artery pseudoaneurysm is a rare disease which most often occur in middle-aged people between 50 and 58 years old, with a male predominance of a male:female ratio of 4.5:1 (5). The age of our patient (76 years old) is older than the usual age of onset, and the female gender of our case is also unique in this male predominant disease. In visceral arteries, generally true aneurysm is more common than pseudoaneurysm. However, in gastroduodenal, pancreaticoduodenal, and superior mesenteric arteries, it is the pseudoaneurysm more frequent (6).

In gastroduodenal artery pseudoaneurysm, the most common causes are pancreatitis (47%), followed by ethanol abuse (25%), and peptic ulcer disease (17%) (5). There are also several cases of GDA pseudoaneurysm appearing several days, weeks, months, or even 1–3 years after surgery or other invasive procedures (7–19). Due to past histories of gastric ulcer, it is likely that peptic ulcer disease is the cause of GDA pseudoaneurysm in our patient.

When a GDA pseudoaneurysm is unruptured, common presentations are abdominal pain, nausea, and vomiting resulting from compression of gastrointestinal tract, as in our case. Moreover, due to the proximity between GDA and the bile duct, a GDA pseudoaneurysm or its associated hematoma can also compress the bile duct and result in obstructive jaundice (20–22). There is even a case of duodenal necrosis from compression by a GDA pseudoaneurysm (23). Also, rare presentations of hyperamylasemia (24) and midline back soreness (25) are reported. When a GDA pseudoaneurysm ruptures, it has a mortality of 40% (5). If a GDA pseudoaneurysm ruptures into the duodenum, the clinical presentations are signs of upper gastrointestinal bleeding such as hematemesis and melena (5). There are also cases the pseudoaneurysm rupture into pancreatic duct and bile duct, leading to hemosuccus pancreaticus (26–28) and hemobilia (16), respectively.

The most frequent imaging modality to detect pseudoaneurysms is CT angiography (CTA), which revealed a contrast filled sac (4). However, the gold standard for diagnosing pseudoaneurysms is digital subtraction angiography (DSA), which is mainly used in conditions when a pseudoaneurysm is highly suspected clinically but is not seen in CTA (4). The pseudoaneurysm appear to be more irregular than true aneurysm, and it also has features of eccentric thrombosis, eccentric location, and saccular in shape (4). Sometimes, it may be difficult to distinguish between a pseudoaneurysm and true aneurysm in imaging (4).

In true aneurysms of visceral arteries, the indications of treatment are symptomatic, size greater than 2 cm, growth rate exceeding 0.5 cm per year, or in women of childbearing age. In pseudoaneurysms, however, regardless of size, all should be managed as soon as possible once detected (29). The reason is that pseudoaneurysms can rupture more easily than true aneurysms (76.3% vs. 3.1%) (6). GDA pseudoaneurysm has a 75% incidence of rupture (30).

Generally, the treatment options for visceral artery aneurysm include open surgery and minimally invasive endovascular treatment. Open surgery is indicated when the patient’s vital signs are unstable or when endovascular treatment has failed (5). It typically involves ligation of arteries proximal and distal to the aneurysm, aneurysmectomy, and reestablishment of vascular network or end organ resection depending on the collateral circulation of the organ as well as the aneurysm location (31). When the patient is hemodynamically stable, with multiple comorbidities, or previous abdominal surgery with possible intraperitoneal adhesion, endovascular treatment is the treatment of choice. Endovascular treatment has advantages of minimal invasiveness, lower morbidity, and better quality of life (32). The endovascular treatments exclude the aneurysm from the circulation and include coil embolization, liquid embolic agents (e.g., NBCA), and stent placement (33). Which endovascular techniques are being performed depends on anatomical factors. Coil embolization is the most commonly used endovascular treatment. It involves embolization of aneurysm as well as proximal and distal arteries, with a potential complication of coil migration when used in large saccular aneurysm or when there is infection, inflammation, or malignancy around the pseudoaneurysm (31, 32, 34). Embolization with liquid embolic agents carries the risk of distal embolization and is therefore more technically challenging. It can be used when the aneurysm is in the terminal branch of arteries or when the vessels are too small and tortuous for coil embolization (31, 35). Stent placement requires adequate vessel length and minimal vessel tortuosity, carrying a risk of stent thrombosis, and is only considered when flow through the artery is essential (32). Our patient was hemodynamically stable in the emergency department. Also, the pseudoaneurysm was in the terminal branch of the gastroduodenal artery. Hence, endovascular therapy using liquid embolic agent NBCA is a reasonable treatment option.

In addition to open surgery and endovascular treatment, there are other treatment options including percutaneous and endoscopic ultrasound (EUS) approaches. Percutaneous thrombin injection is used when the pseudoaneurysm cannot be accessed by endovascular means (4). It involves direct injection of thrombin into the pseudoaneurysm under ultrasound, CT, or fluoroscopy guidance, resulting in thrombosis of the pseudoaneurysm (29). The drawback of this technique is that thrombin is not radiopaque and hence may cause undetectable distal embolization. An endoscopic ultrasound approach is only used when the pseudoaneurysm can be detected on EUS, and thrombin or other embolic agents are injected into the pseudoaneurysm under EUS guidance (4). Also, using over-the-scope-clip to treat active bleeding from GDA pseudoaneurysm has also been reported (36).

The society for vascular surgery published a clinical practice guideline on the management of visceral artery aneurysms in 2020. For the gastroduodenal artery, there is a strong recommendation for using coli embolization whether the aneurysm is ruptured or unruptured (35). As for the liquid embolic agent used in our case, it is weakly recommended in the guideline (35). This is consistent with the literature in the last two decades. In the medical literature regarding GDA pseudoaneurysms from 2001 to 2022, there are 30 cases that have been treated with endovascular embolization, 5 cases treated with open surgery, 2 cases treated with percutaneous embolization, 1 case treated with EUS-guided embolization, and 2 cases without any treatment (Table 1). These treatment strategies were all successful except for 2 failed cases in endovascular embolization (case 11 and case 23). Both initially presented with hypovolemic shock and subsequently managed successfully with open surgery. The success rate of endovascular therapy is comparable to open surgery (93.3% vs. 100%). Among the 30 cases of endovascular embolization, 23 cases are managed with coil embolization, 2 cases with stent placement, 3 cases with liquid embolic agent NBCA combined with coil embolization, 1 case with polyvinyl alcohol (PVA) particles, and 1 case with an unknown endovascular method. To the best of our knowledge, there was no patient of GDA pseudoaneurysm treated with liquid embolic agent NBCA alone. However, we demonstrated such a successful case using NBCA alone in the elder female patient with GDA pseudoaneurysm.

**Table 1:** GDA pseudoaneurysms in medical literatures from 2001 to 2022.

#	Authors	Age and gender of patient	Clinical presentation	Related to surgery or other invasive procedure	First treatment	Success of first treatment
1	Rancaño et al. (7)	48, Male	Epigastric pain	Yes	Endovascular embolization with coils	Yes
2	João et al. (8)	58, Male	Melena	Yes	Endovascular embolization with coils	Yes
3	Stravodimos et al. (9)	71, Male	Upper GI and intra-abdominal bleeding	Yes	Endovascular embolization	Yes
4	Miyazawa et al. (10)	71, Male	-	Yes	Endovascular stent placement	Yes
5	Zarin et al. (11)	18, Male	Melena	Yes	Endovascular embolization with PVA particles	Yes
6	Kunitomo et al. (12)	54, Male	Bloody stool	Yes	Endovascular embolization with coils & NBCA	Yes
7	Awada et al. (13)	56, Male	Hematemesis and melena	Yes	Surgery with vessel ligation	Yes
8	Young et al. (14)	80, Female	Epigastric pain, nausea, and vomiting	Yes	Endovascular embolization with coils	Yes
9	Phelps et al. (15)	55, Male	Recurrent bleeding	Yes	Percutaneous embolization with coils	Yes
10	Kurniawan et al. (16)	44, Female	Hemobilia	Yes	Surgery with ligation and excision	Yes
11.	Kim et al. (17)	73, Male	Abdominal distention and hypovolemic shock	Yes	Endovascular embolization with coils	No
12	Loveček et al. (18)	58, Male	Epigastric pain	Yes	Endovascular stent placement	Yes
13	Macedo et al. (19)	77, Male	Hematemesis and hematochezia	Yes	Endovascular embolization	Yes
14	Casas Deza D et al. (20)	74, Female	Jaundice	No	Endovascular embolization with coils	Yes
15	Kossak et al. (21)	35, male	Jaundice	No	Surgery with ligation	Yes
16	Sharma et al. (37)	50, Male	Melena	No	EUS-guided coil embolization and thrombin injection	Yes
17	Budzyński et al. (38)	42, Female	GI bleeding	No	Endovascular embolization with coils	Yes
18	Chang et al. (30)	71, Male	Bloody stool	No	Endovascular embolization with coils	Yes
19	Suzuki et al. (39)	65, Male	Hematemesis	No	Endovascular embolization with coils	Yes
20	Sinduja et al. (40)	70, Male	Hematemesis and melena	No	Endovascular embolization with coils	Yes
21	Volpi et al. (41)	69, Male	Hematemesis and melena	No	Endovascular embolization with coils	Yes
22	Nouira et al. (42)	40, −	Epigastric pain and hematemesis	No	No treatment (death before treatment)	-
23	Kohama et al. (43)	85, Male	Hematemesis and hypovolemic shock	Yes	Endovascular embolization	No
24	Elazary et al. (44)	18, Female	UGI bleeding	Yes	Endovascular embolization with coils	Yes
25	Ahmed et al. (45)	56, Female	Epigastric pain	No	Endovascular embolization with coils	Yes
26	Saqib et al. (26)	68, Male	Abdominal pain	No	Endovascular embolization with coils	Yes
27	Cui et al. (27)	39, Male	Hematemesis and melena	No	Endovascular embolization with coils and NBCA	Yes
28	Lacey et al. (28)	62, Male	Melena	No	Endovascular embolization	Yes
29	Huang et al. (25)	54, Male	Midline back soreness	No	No (refuse operation)	-
30	Singh et al. (46)	35, Male	Epigastric pain and blood-stained vomiting	No	Surgery with ligation	Yes
31	Shrestha et al. (47)	45, Female	Epigastric pain and vomiting	No	Endovascular embolization with coils	Yes
32	Chen et al. (23)	55, Male	Epigastric pain	No	Surgery	Yes
33	Smith et al. (48)	64, Male	Abdominal pain	No	Endovascular embolization with coils	Yes
34	Lim et al. (49)	39, Female	Abdominal pain	No	Endovascular embolization with coils and NBCA	Yes
35	Leow et al. (50)	53, Male	Epigastric pain, melena, and hematemesis	No	Endovascular embolization with coils	Yes
36	Geoghegan et al. (51)	24, Male	Abdominal pain	No	Percutaneous thrombin injection	Yes
37	Sgantzou et al. (52)	64, Male	Hematemesis	Yes	Endovascular embolization with coils	Yes
38	Galanakis (24)	79, Male	Abdominal pain	No	Endovascular embolization with coils	Yes
39	Klauß et al. (53)	47, Male	Acute upper abdominal pain	No	Endovascular embolization with coils	Yes
40	Shell et al. (22)	46, Female	Abdominal pain, nausea, and hematemesis	No	Endovascular embolization with coils	Yes

In general, GDA pseudoaneurysm is an uncommon disease that should be managed as soon as possible given the high risk of rupture. Endovascular therapy with coil embolization is the most recommended treatment in the hemodynamic stable patient with such a condition. However, we present a case of GDA pseudoaneurym managed with endovascular embolization using liquid embolic agent NBCA alone, a treatment strategy that has not been used in such a condition. The successful experience of our case may provide a new treatment strategy in patients with GDA pseudoaneurysm.

## Data availability statement

The original contributions presented in the study are included in the article/supplementary material, further inquiries can be directed to the corresponding author.

## Ethics statement

Written informed consent was obtained from the participant/patient(s) for the publication of this case report.

## Author contributions

All authors listed have made a substantial, direct, and intellectual contribution to the work and approved it for publication.

## Conflict of interest

The authors declare that the research was conducted in the absence of any commercial or financial relationships that could be construed as a potential conflict of interest.

## Publisher’s note

All claims expressed in this article are solely those of the authors and do not necessarily represent those of their affiliated organizations, or those of the publisher, the editors and the reviewers. Any product that may be evaluated in this article, or claim that may be made by its manufacturer, is not guaranteed or endorsed by the publisher.
